# Total plasma magnesium, zinc, copper and selenium concentrations in obese patients before and after bariatric surgery

**DOI:** 10.1007/s10534-022-00368-7

**Published:** 2022-02-09

**Authors:** Stephen J. Hierons, Anthony Catchpole, Kazim Abbas, Wingzou Wong, Mathew S. Giles, Glenn V. Miller, Ramzi A. Ajjan, Alan J. Stewart

**Affiliations:** 1 School of Medicine, University of St Andrews, Medical and Biological Sciences Building, St Andrews, Fife UK; 2 Scottish Trace Element and Micronutrient Diagnostic and Reference Laboratory, Department of Biochemistry, NHS Greater Glasgow and Clyde, Glasgow, UK; 3 Renal Transplant Unit, Manchester Royal Infirmary, Manchester, UK; 4 Endoscopy and GI Physiology Unit, York Hospital, York, UK; 5 Leeds Institute of Cardiovascular and Metabolic Medicine, University of Leeds, Leeds, UK

**Keywords:** ICP-MS, Metal homeostasis, Obesity, Roux-en-Y surgery, Zinc/copper ratio

## Abstract

Obesity enhances the risk of type-2 diabetes, cardiovascular disease and inflammatory conditions and often leads to metal dyshomeostasis, which contributes to the negative health aspects associated with the disease. In severe cases, bariatric surgery can be recommended to achieve sustained weight loss and improvement in health. Here, magnesium, zinc, copper and selenium concentrations were examined in 24 obese patients (7 males; 17 females) before and 9 months after undergoing Roux-en-Y gastric bypass surgery. All patients lost weight over this period, with the mean BMI reducing from 51.2±7.1 kg/m^2^ to 37.2±5.5 kg/m^2^. Moreover, whole-blood glycated haemoglobin (HbA1c), as a marker of average glycaemia, was also measured and a correlative analysis of this parameter with metal concentrations performed. Significant alterations in the plasma concentrations of magnesium, zinc (both increased by 13.2% and 25.2% respectively) and copper (decreased by 7.9%) were observed over this period (plasma selenium concentration was unchanged), with BMI values correlating with plasma magnesium (p = 0.004) and zinc (p = 0.022) concentrations. At 9 months post-surgery, an increase in mean zinc/copper ratio was observed (0.86±0.29 compared to 0.63±0.14 pre-surgery). Comparison of whole-blood HbA1c concentrations pre- and post-surgery revealed a reduction from 6.50±1.28% pre-surgery to 5.51±0.49% post-surgery. Differences in plasma HbA1c and magnesium at either pre- and post-surgery correlated significantly, as did HbA1c and magnesium levels when pre- and post-surgery values were analysed together. Collectively, this work reveals that bariatric surgery, in conjunction with lifestyle/dietary changes, lead to improvements in the nutritional status of magnesium, zinc and copper. Furthermore, the observed improvements in magnesium and zinc were associated with weight loss and in the case of magnesium, to better glycaemic control.

## Introduction

Obesity is a complex disorder associated with excessive body fat that presents a risk to health. A person is defined as being obese when their body mass index (BMI) is 30 kg/m^2^ or above. The worldwide prevalence of obesity has increased over the past 50 years, to reach what are described to be pandemic levels (Blüher 2019), and is caused by a combination of genetic, behavioural, socioeconomic and environmental factors (Hruby and Hu 2015). Obese individuals generally have an increased risk of co-morbidities including type-2 diabetes, hypertension, dyslipidemia, cardiovascular disease, stroke, gall bladder disease, osteoarthritis and gout (Khaodhiar et al. 1999). Although obesity is associated with excessive dietary consumption, it is also associated with micronutrient abnormalities (Via 2012; Lapik et al. 2020). These can be defined as deficiencies or excessive amounts of certain nutrients in the body that include essential metal ions such as Mg^2+^, Zn^2+^ and Cu^2+^. For example, obesity is associated with elevated serum levels of copper (Yang et al. 2019) and reduced plasma levels of magnesium (Piuri et al. 2021), zinc (Di Martino et al. 1993) and selenium (Beckett and Arthur 2005). Obesity has also been implicated as a risk factor for iron deficiency (Aigner et al. 2014). Current evidence suggests that nutrient abnormalities contribute to the development of the metabolic defects, inflammatory disorders and thrombotic complications associated with the disease. For example, obesity-related hypomagnesaemia is associated with insulin resistance, atherosclerosis, myocardial infarction, hypertension and renal calculi (Nadler and Rude 1995; De Leeuw et al. 1992; Huerta et al. 2005). Poor zinc status in obesity is associated with inflammation, oxidative stress and both lipid and glucose metabolism (Olechnowicz et al. 2017), with zinc supplementation shown to improve body weight management (Khorsandi et al. 2019), potentially through regulation of leptin levels (Liu et al. 2013). Furthermore, obesity is associated with serum copper elevations (~15% higher than controls), with serum copper positively correlating with leptin, insulin, and leptin/BMI ratio (Yang et al. 2019).

Treatment of obesity generally includes recommending patients make lifestyle alterations, such as dietary changes and increasing exercise. In some cases, medications that reduce appetite or alter the absorption of fat in the gut (e.g. glucagon-like peptide receptor agonists and lipase inhibitors) are also prescribed. However, these generally have a high cost and are often associated with unwanted side effects and only relatively modest weight loss (Tak and Lee 2021). In severely obese individuals (BMI > 40 kg/m^2^), or those with milder obesity (BMI of 35–40 kg/m^2^) in addition to other significant disease, surgical procedures often represent the best strategies for intervention. These can be recommended when non-surgical measures have been attempted but the person has either not achieved, or been able to maintain clinically beneficial weight loss. An individual must also be assessed as fit for anaesthesia/surgery and commit to long-term follow-up and lifestyle changes (National Institute for Health and Care Excellence 2014). In such cases, the most common surgical procedure employed is the Roux-en-Y gastric bypass; a laparoscopic surgery that reduces the effective size of the stomach by 80%. It is effective in promoting sustained weight loss in obese patients, with consequent reductions in the incidence and severity of co-morbidities (Sharples and Mahawar 2020), and an increase in life expectancy, particularly in those with diabetes at the time of surgery (Lent et al. 2017).

Mechanistically, the improvements in health status offered by gastric bypass surgery in obese individuals will likely be due, in part, to improvements in nutrient status. In this study, we examine how bariatric surgery (Roux-en-Y), and associated dietary recommendations influence total plasma concentrations of magnesium, copper, zinc and selenium in patients before and 9 months after surgery. We also explore whether potential relationships exist between the plasma concentrations of these nutrients and both BMI and glycaemic status.

## Methods

### Sample collection and treatment

A total of 24 Roux-en-Y surgical patients (17 females and 7 males) were recruited from York Hospital, York, United Kingdom. Male or female patients over the age of 18 referred to the hospital for gastric bypass surgery for obesity were included. Patients were excluded if they were under 18 years old, were diagnosed with type 1 diabetes or endocrine disorders other than type 2 diabetes, had a history of alcohol or drug abuse, had a significant psychological history, had history of deep vein thrombosis or pulmonary embolism, were taking warfarin, were pregnant, developed post-operative complications or had a history of active malignancy. With the exception of one participant (who was of Asian-British decent), all participants were Caucasians. Plasma samples were collected following approval by the National Research Ethics Service Committee Yorkshire & The Humber - Sheffield (REC reference: 11/H1308/16) after obtaining written informed consent. Two weeks before surgery patients were placed on a low-calorie/low-fat diet, containing 800–1000 kcal/day. At this point, they commenced taking an over the counter multi-vitamin pill until 4 weeks post-surgery. After surgery patients were given a puree diet for the first 4 weeks (i.e. yoghurt, soup, fruit juice, oatmeal, cottage cheese, mashed potato, scrambled egg, pureed meat or baby food). They were then either prescribed Forceval multivitamin (one capsule daily; which contains 12 vitamins and 12 minerals including copper (2 mg), magnesium (30 mg), selenium (50 µg) and zinc (15 mg) or advised to take another complete multi-vitamin and mineral supplement of their choosing. Other supplements given post-operatively after 4 weeks were ferrous fumarate (210 mg twice a day for menstruating women or once daily for non-menstruating women and men), ferrous gluconate (300 mg once daily), Adcal D3 (vitamin D supplement; 1 tablet twice daily), lansoprazole FASTAB (30 mg once daily as an antacid to relieve symptoms of overproduction of acid in the stomach; for those with intolerance, omeprazole 40 mg was given once daily instead) and vitamin B12 injection (1 mg intramuscular injection every 3 months). For all subjects, blood samples were collected in both lithium heparin and EDTA tubes before surgery (no more than 48 h before the operation) and not before nutritional intervention and at a 9-month (±1 week) follow-up appointment after surgery. EDTA-treated whole blood was used to measure HbA1c by the Laboratory Medicine service at York Hospital using a boronate affinity chromatography-based method (Little and Roberts 2009). An HbA1c concentration of <6.0% (<42 mmol/mol) was deemed normoglycaemic as per World Health Organisation criteria. Plasma was separated within 2 h of collection by centrifugation at 2400 × g for 20 min at 4 °C, snap-frozen in liquid nitrogen and stored at − 80 °C until analysis.

### Inductively-coupled plasma-mass spectrometry (ICP-MS)

Single element stock solutions (Centripure, Merck, UK) traceable to the National Institute of Standards and Technology, USA (NIST) were used as calibration and internal standards. All standards were prepared in 2% v/v butan-1-ol (Sigma Aldrich, Poole, UK), 0.05% w/v EDTA (Sigma Aldrich), 0.05% v/v Triton-X-100 (Sigma Aldrich), 1% v/v ammonia (Romil, Cambridge, UK) in 18.2 MΩ deionised water (Elga Maxima, High Wycombe, UK). Plasma samples (collected in lithium heparin tubes) were diluted 1 in 10 with the same solution used to prepare standards with 25 ppb germanium (Sigma-Aldrich; 1000 mg/L stock). ClinChek® 1 & 2 (RECIPE Chemicals, Munich, Germany) and Seronorm™ 1 (SERO, Billingstad, Norway) human serum certified reference materials were used to demonstrate accuracy. ClinChek®1 and 2 are materials with consensus values while Seronorm™ is traceable to a NIST-certified reference material.

Magnesium, copper, zinc and selenium analyses were performed simultaneously using an Agilent 7900 ORS-ICP-MS (Agilent Technologies, Santa Clara, California, USA), controlled using Mass Hunter software (version 4.1, Agilent Technologies). Argon was used to form the plasma (99.9% pure; CryoServices Ltd, Worcester, UK). Polyatomic interferences for magnesium, copper, zinc and selenium were removed through collision-induced dissociation and kinetic energy discrimination using helium gas (99.9% pure; Air Products and Chemicals, Pennsylvania, USA). The concentration of all elements was measured three times within a single run using the central 0.05 m/z of the peak. The ICP-MS was equipped with a CETAC ASX-500 series autosampler (Teledyne CETAC, Omaha, USA), an Integrated Sample Introduction System and Discrete Sampler-3 (ISIS-DS, Agilent Technologies) and a G32992A re-circulating chiller (Polyscience, Illinois, USA). The ISIS-DS was fitted with a Quartz Scott-type, double-pass spray chamber (Agilent Technologies), a glass micro-mist nebuliser (Agilent Technologies) and a sample loop. The sample loop (0.63 cm^3^ volume) was prepared in-house using 0.8 mm internal diameter and polytetrafluoroethylene sample tubing (Agilent Technologies). A quartz torch with a 2.5 mm internal diameter was used (Agilent Technologies). Nickel sampling and skimmer cones were always used (Agilent Technologies). Instrument parameters were optimised each day by performing an auto-tune while aspirating a tuning solution containing 10 ppb of lithium, yttrium, cobalt, cerium and thallium. Typical instrument parameters were as follows: isotopes monitored were ^24^Mg, ^63^Cu, ^66^Zn, and ^78^Se; radio frequency (RF) power = 1550 W; RF matching = 1.70; sampling depth = 10 mm; carrier gas = 1.05 L/min; make up gas = 0 L/min; spray chamber temperature = 2 °C; helium octopole reaction system flow = 5.0 mL/min; nebuliser pump = 0.1 rps. Three certified reference materials (Seronorm 1 and ClinCheck 1 and 2) with assigned values and ranges were used to demonstrate the accuracy of the method used to determine the concentrations of Mg, Cu, Zn and Se in human plasma (Table 1). Reference materials were each measured 6 times. The mean for all measurands were within the assigned range and had satisfactory recoveries ranging between 92.9 and 105.8%. The precision of the method, represented by the coefficient of variation (CV), was also satisfactory with values less than or equal to 4.3%.

**Table 1:** Accuracy of the ICP-MS method and details of the concentration of trace elements in each standard reference material (coefficient of variation and recovery)

Measurand	Specimen	Certified value (µmol/L)	Confidence interval (µmol/L)	Mean concentration (from 6 determinations; µmol/L)	CV (%)	Recovery (%)
Plasma Mg	Seronorm1	690	550–830	680	4.3	98.9
	ClinCheck 1	640	580–710	600	2.5	93.1
	ClinCheck 2	1210	1090–1330	1120	0.7	92.9
Plasma Cu	Seronorm1	17.1	15.7–18.5	17.0	3.8	99.4
	ClinCheck 1	10.9	8.7–13.1	10.8	2.0	98.6
	ClinCheck 2	19.1	15.4–23.0	19.1	0.5	99.8
Plasma Zn	Seronorm1	16.8	14.6–19.0	16.3	4.0	97.0
	ClinCheck 1	17.7	14.2–21.3	17.6	2.2	99.4
	ClinCheck 2	23.2	18.8–28.3	24.5	0.3	105.8
Plasma Se	Seronorm1	1.10	0.96–1.25	1.08	3.8	97.9
	ClinCheck 1	1.04	0.83–1.24	1.04	1.9	100.4
	ClinCheck 2	1.52	1.22–1.82	1.54	0.8	101.1

### Statistical analysis and representation

Data are presented as mean ± standard deviation (SD). Graphs were generated and statistical analysis was performed using Prism 9.0 (GraphPad Software, La Jolla, CA). Data were distributed normally and differences between groups were analysed using multiple Student’s t-tests (paired or non-paired as appropriate), while correlations between linear variants were analysed with Pearson’s correlation. Significance threshold in all cases was set at p≤0.05.

## Results and discussion

### Anthropometric characteristics, whole-blood HbA1c and plasma zinc, copper, magnesium, selenium concentrations and zinc/copper ratios in all subjects before and 9 months after bariatric surgery

**Fig. 1:**
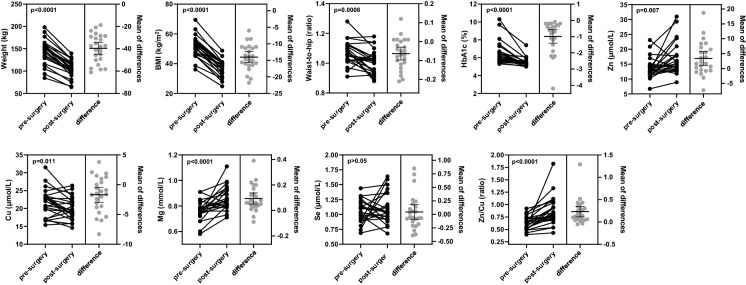
Comparison between mean anthropometric values, whole-blood HbA1c and trace element concentrations in patients before and 9 months after bariatric surgery. Panels on the left show individual values from patients before (pre-surgery) and 9 months after (post-surgery) Roux-en-Y bariatric surgery. Panels on the right show differences in values between each patient pre- and post-surgery. Error bars represent the mean of differences +/- SEM

Anthropometric values (including weight, BMI, waist/hip/neck circumferences and waist-to-hip ratio) and whole-blood concentrations of HbA1c and plasma trace elements were measured and compared in the 24 patients before and 9 months after Roux-en-Y gastric bypass surgery. Table 2 summarises the demographic characteristics of the cohort as well as anthropometric values and mean HbA1c and plasma trace element concentrations in each group. Figure 1 shows a graphical comparison of mean anthropometric values and whole-blood HbA1c and plasma trace element concentrations in the patients before and 9 months after bariatric surgery.

**Table 2:** Demographic characteristics of the studied population and mean anthropometric values and whole-blood HbA1c and trace element concentrations before and 9 months after surgery

Characteristics	Values			
Age at recruitment (years ± SD)	45.6 ± 10.0			
% Males (number)	29.2 (7/24)			
	Pre-surgery	9 months post-surgery	p-value	Significance
Weight (kg ± SD)	150.9 ± 34.9	106.4 ± 24.5	<0.0001	****
BMI (kg/m^2^ ± SD)	51.2 ± 7.1	37.2 ± 5.5	<0.0001	****
Waist circumference (cm ± SD)	143.4 ± 11.4	117 ± 12.2	<0.0001	****
Hip circumference (cm ± SD)	135.8 ± 11.5	116 ± 10.9	<0.0001	****
Waist-to-hip (ratio ± SD)	1.07 ± 0.08	1.01 ± 0.08	0.0008	***
Neck circumference (cm ± SD)	45.6 ± 5.05	40.0 ± 4.5	<0.0001	****
HbA1c mean (% ± SD)	6.50 ± 1.28	5.51 ± 0.49	<0.0001	****
% of individuals with Dysglycaemia (number)	54.2 (11/24)	8.3 (2/24)	N/A	N/A
% of individuals prescribed metformin (number)	29.2 (7/24)	8.3 (2/24)	N/A	N/A
% of individuals prescribed a statin (number)	25.0 (6/24)	20.8 (5/24)	N/A	N/A
Zinc conc. mean (µmol/L ± SD)	13.5 ± 3.5	16.9 ± 5.4	0.007	**
Copper conc. mean (µmol/L ± SD)	21.6 ± 3.9	19.9 ± 3.1	0.011	*
Magnesium conc. mean (mmol/L ± SD)	0.76 ± 0.08	0.86 ± 0.09	0.0002	****
Selenium conc. mean (µmol/L ± SD)	1.05 ± 0.17	1.09 ± 0.26	0.498	ns
Zinc/copper (ratio ± SD)	0.63 ± 0.14	0.86 ± 0.29	<0.0001	****

Dysglycaemia was defined by a HbA1c concentration >6.0%. The p-values were calculated using paired T-test. Statistical significance is indicated as: ns, not significant (p>0.05), * for p≤0.05, ** for p≤0.01, *** for p≤0.001 and **** for p≤0.0001. N/A, not applicable

Over the period, the mean weight of the cohort reduced by 29.5% from 150.9±34.9 kg pre-surgery to 106.4±24.5 kg post-surgery (p < 0.0001) and the BMI by 28.3% from 51.2±7.1 kg/m^2^ pre-surgery to 37.2±5.5 kg/m^2^ (p < 0.0001). Significant differences were also observed in the waist, hip and neck circumference, with the waist-to-hip ratio decreasing from 1.07±0.08 to 1.01±0.08 (p < 0.0001). These measurements indicate the treatment was successful in promoting substantive weight loss by 9 months post-surgery. The mean whole-blood HbA1c concentration reduced from 6.50±1.28% to 5.51±0.49% (p < 0.0001), suggesting that the intervention led to improved glycaemic control in the cohort. Indeed, 11 of the patients (54.2%) were dysglycaemic (defined as an HbA1c concentration ≥6.0%) before surgery, while only 2 (8.3%) remained dysglycaemic 9 months after surgery. Unsurprisingly, a greater reduction in HbA1c concentration was observed in those with dysglycaemia before surgery. Prior to surgery, 7 of the participants were prescribed metformin and 6 were prescribed a statin. At 9-months post-surgery, 2 were still prescribed metformin (the same 2 individuals who remained dysglycaemic) and 5 were still taking a statin.

Significant differences in the mean plasma concentrations of zinc (increase from 13.5±3.5 µmol/L to 16.9±5.4 µmol/L; p-value=0.007), copper (decrease from 21.6±3.9 µmol/L to 19.9±3.1 µmol/L; p = 0.011) and magnesium (increase from 0.76±0.08 mmol/L to 0.86±0.09 mmol/L; p < 0.0001) were observed 9 months after surgery. Of note, a decrease in copper levels was evident even given supplement therapy with this metal. No difference was observed in mean plasma selenium concentration post-surgery over this period. Also, no significant differences were observed in the plasma concentrations of zinc, magnesium, copper or selenium between dysglycaemic and normoglycaemic individuals either before surgery or 9 months after surgery.

The normal plasma reference intervals for the four analysed trace elements are shown in Table 3. Whilst the mean values of these metals were all within their respective reference intervals both before and after surgery, the respective changes in magnesium and copper post-surgery generally altered the mean concentration towards the centre of the normal range. This is likely indicative of improved homeostatic regulation of these trace elements. It is worthy of note that of the 24 subjects, one was found to have magnesium below the normal range, and one had both magnesium and zinc levels below the normal range, whilst two (not including the same patients) had copper levels higher than the normal range prior to surgery. At 9 months after surgery, the levels of magnesium and zinc in the patients with lower-than-normal levels returned to values within the normal range as did the copper concentration in the two individuals who previously had hypercupraemia. At 9 months post-surgery, one individual had magnesium levels slightly above the normal range, two were higher than normal for copper and four were higher than normal for zinc, potentially due to over-supplementation. Hypermagnesaemia (defined as plasma concentrations >1.05 mmol/L), which was observed in one patient, is associated with hypotension, respiratory depression and cardiac arrest (Nishikawa et al. 2018). Hypercupraemia can lead to nausea, stomach pain, hypotension and jaundice (Kim et al. 2019). Abnormally high zinc levels, which we observed in 5 of the 24 patients enrolled in the study (20.8%), can lead to a range of symptoms including nausea, stomach pain, diarrhoea, flu-like symptoms, hypogeusia and copper deficiency (Plum et al. 2010; Science et al. 2012). The zinc/copper ratios were also calculated with the mean ratios found to increase from 0.63±0.14 pre-surgery to 0.86±0.29 at 9 months post-surgery (p < 0.0001). The normal zinc/copper (Zn/Cu) ratio, in children and adults should be ~1.0 (Böckerman et al. 2016), suggesting that treatment on average led to a significant improvement in this ratio in the cohort. However, one of the patients found to have abnormally high zinc levels after treatment exhibited a zinc/copper ratio of 1.82 at 9 months post-surgery. The molar ratio of these metals, rather than the absolute amount of either in the body alone, is thought to enable optimal enzyme functioning (Osredkar and Sustar 2001). This is due to the vastly differing abilities of these metals to catalyse specific chemical reactions while part of the active site of an enzyme, combined with an overlap in their propensity under pathophysiological concentrations to co-ordinate at such sites. It is worthy of note that some of the participants were prescribed metformin. It has been suggested that metformin can bind some metals (such as copper; Logie et al. 2012). However, given that only a relatively small proportion of the patients were on this type of medication there is no evidence to suggest that this impacted significantly on the results obtained.

**Table 3:** Normal reference intervals for plasma magnesium, zinc, copper and selenium

Trace Element	Reference interval	Reference
Magnesium	0.65–1.05 mmol/L	Ryan (1991)
Zinc	11–18 µmol/L for males aged >9 years 10–18 µmol/L for females aged >9 years	Hotz et al. (2003)
Copper	10–22 µmol/L for males 11–25 µmol/L for females	Abbassi-Ghanavati et al. (2009)
Selenium	0.75–1.50 µmol/L for adults aged ≥17 years	Public Health England (2014)

### Differences in anthropometric characteristics and whole-blood HbA1c and plasma trace element concentrations between males and females

**Fig. 2:**
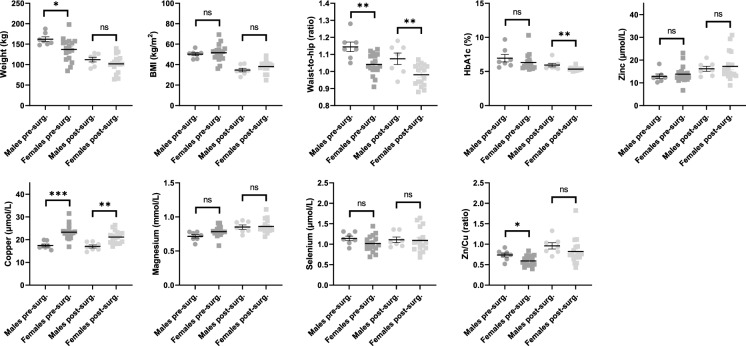
Comparison between mean anthropometric values, whole-blood HbA1c and trace element concentrations in males and females before and 9 months after surgery. Statistical significance is indicated as: ns, not significant (p > 0.05), * for p ≤ 0.05, ** for p ≤ 0.01 and *** for p ≤ 0.001

A comparison of mean anthropometric values and mean whole-blood HbA1c and plasma trace element concentrations pre- and post-surgery between males and females was carried out (Table 4; Fig. 2). Prior to surgery, the mean weight of the males was higher than the females (162.2±14.8 kg compared to 137±28.4 kg; p = 0.038). Males on average lost more weight (p = 0.007). However, 9 months after surgery there was no statistical difference in weight between males and females. The mean BMI values were not significantly different between sexes either before or after surgery. Waist-to-hip ratios were higher in males both before and 9 months after surgery (1.14±0.07 compared to 1.04±0.06 pre-surgery; p = 0.002 and 1.07±0.09 compared to 0.98±0.06 post-surgery; p = 0.007). Whole-blood HbA1c concentrations did not differ between sexes before surgery, but females had a significantly lower HbA1c concentration than males 9 months after surgery (p = 0.009), indicative of a greater mean improvement in glycaemic control. Significant sex-specific differences in the mean plasma concentrations of magnesium (p = 0.034) and copper (p < 0.0001) were also observed before surgery, with the concentrations of both metals higher in females. Females are known to have higher plasma/serum levels of copper than men potentially due to a higher level of absorption in the gut (Johnson et al. 1992; Quinn et al. 2011). Interestingly, studies have shown that chronic oestrogen administration is associated with increased serum copper and ceruloplasmin concentration (Yang et al. 2019). These effects may explain the sex-specific differences observed. The pre-surgery mean zinc/copper ratio was also lower in females (p = 0.019). The mean copper concentration remained higher in females 9 months after surgery (p = 0.002). However, no statistically significant difference in the mean zinc/copper ratio between sexes was observed at 9 months post-surgery.

**Table 4:** Comparison between mean anthropometric values and whole-blood HbA1c and trace element concentrations 9 months after surgery (compared to pre-surgery values) in males and females

	Pre-surgery				9 months post-surgery			
Characteristics	Males	Females	p = value	Significance	Males	Females	p-value	Significance
Weight (kg ± SD)	162.2 ± 14.8	137.0 ± 28.4	0.038	*	112.2 ± 14.9	101.7 ± 21.3	0.251	ns
BMI (kg/m^2^ ± SD)	50.1 ± 3.9	51.6 ± 8.2	0.667	ns	34.7 ± 4.7	38.2 ± 5.7	0.185	ns
Waist-to-hip (ratio ± SD)	1.14 ± 0.07	1.04 ± 0.06	0.002	**	1.07 ± 0.09	0.98 ± 0.06	0.007	**
HbA1c mean (% ± SD)	6.95 ± 1.46	6.32 ± 1.20	0.312	ns	5.93 ± 0.69	5.35 ± 0.23	0.009	**
Zinc conc. mean (µmol/L ± SD)	12.8 ± 2.6	13.8 ± 3.8	0.549	ns	16.2 ± 3.0	17.2 ± 6.2	0.067	ns
Copper conc. mean (µmol/L ± SD)	17.5 ± 1.7	23.3 ± 3.2	0.0003	***	17.0 ± 1.8	21.1 ± 2.7	0.002	**
Magnesium conc. mean (mmol/L ± SD)	0.71 ± 0.06	0.78 ± 0.08	0.034	ns	0.85 ± 0.08	0.86 ± 0.10	0.834	ns
Selenium conc. mean (µmol/L ± SD)	1.14 ± 0.15	1.02 ± 0.20	0.153	ns	1.11 ± 0.18	1.09 ± 0.29	0.897	ns
Zinc/copper (ratio ± SD)	0.74 ± 1.29	0.59 ± 1.2	0.020	*	0.96 ± 0.21	0.82 ± 0.32	0.323	ns

The p-values were calculated using T-test (non-paired). Statistical significance is indicated as: ns, not significant (p>0.05), * for p≤0.05, ** for p≤0.01 and *** for p≤0.001

### Relationship between plasma metal concentrations and BMI

**Fig. 3:**
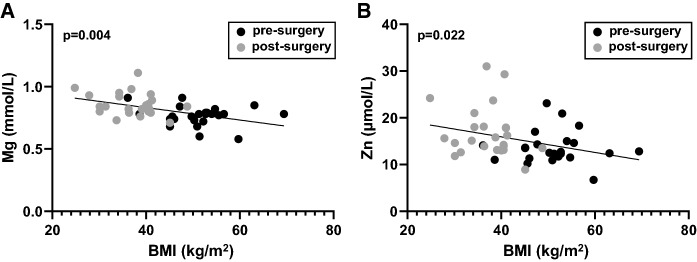
Relationship between BMI value and plasma magnesium and zinc concentrations in all patients before and 9 months after bariatric surgery. Significant correlations were observed between** A** BMI and plasma magnesium concentration (p = 0.004) and** B** between BMI and plasma zinc concentration (p = 0.031) in the pre- and post-surgery samples

Plasma concentrations of magnesium, copper, zinc and selenium from individual patients were compared to respective BMI values measured either before or 9 months after surgery. No statistically significant correlation between pre-surgery values or between the post-surgery values were observed (where p≤0.05 is considered significant). Similarly, no correlation was observed when differences in plasma metal concentrations between pre- and post-surgery values were compared to respective differences in BMI values in individual patients (potentially due to a lack of power). However significant correlations were observed between BMI values and both plasma magnesium (p = 0.004) and zinc (p = 0.031) concentrations when both pre-and post-surgery values of these parameters across individuals were compared together in all patients (Fig. 3). It is possible that these correlations are to a certain degree a consequence of the nutrient supplementation regime provided for the patients after surgery. Although the supplements were designed to replace those nutrients that would be missing from their diet due to reduced food intake, it is perhaps likely that the dietary changes and vitamin/mineral supplementation post-surgery represented a better nutrient balance in the diet than the patients had before surgery. In addition, it has been reported that the gut microbiota can be influenced by Roux-en-Y surgery, which may impact upon metal ion homeostasis (Lau et al. 2021; Pajarillo et al. 2021). However, an observed correlation between plasma magnesium and BMI is consistent with studies that have found obesity to be associated with lower serum/plasma levels of magnesium (De Leeuw et al. 1992; Nadler and Rude 1995; Huerta et al. 2005; ul Hassan et al., 2017). It has also been suggested that dietary magnesium intake is associated with a lower BMI (Castellanos-Gutiérrez et al. 2018). The data suggest that the reduced levels of magnesium in obesity are reversible with weight loss and that they may exhibit a linear relationship with BMI. It is not possible to conclude exactly what the mechanisms underlying this potential relationship are, but it has been suggested that elevated leptin levels, as are associated with obesity (Obradovic et al. 2021), can influence the urinary excretion of magnesium potentially leading to a reduction in circulating magnesium levels (Atabek et al. 2006). As with plasma magnesium, reductions in plasma zinc levels are also known to be associated with obesity (Atkinson et al. 1978; Di Martino et al. 1993; Tungtrongchitr et al. 2003; Marreiro et al. 2004; Zaky et al. 2013), consistent with the correlation between BMI and plasma zinc observed here. Several mechanistic factors have been implicated in this relationship. These include (over-)consumption of energy-rich foods high in phytate, which prevents absorption of zinc in the gut (Ho et al. 2016) and increased excretion of zinc (Martins et al. 2014) – note these factors may be linked. Reduced levels of serum albumin, the protein primarily responsible for binding and transporting the zinc in the circulation (Handing et al. 2016), are associated with obesity (Mosli and Mosli 2017), where the plasma concentration was reported to be 38.0 g/L in individuals of normal weight and 34.6 g/L and 33.8 g/L in obese and morbidly obese individuals, respectively. In addition, it has been reported that the expression of certain cellular zinc transporters in tissues including adipose, brain and leukocytes can be affected by obesity, which can lead to zinc dyshomeostasis in various tissues (Noh et al. 2014; Olesen et al. 2016; Troche et al. 2016; Maxel et al. 2017), potentially impacting on plasma zinc concentration.

### Relationship between plasma metal concentrations and HbA1c

**Fig. 4:**
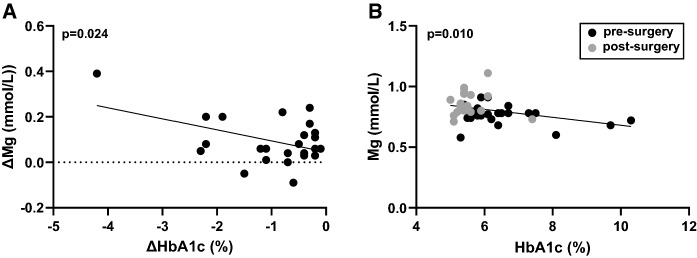
Relationships between whole-blood HbA1c and plasma magnesium concentrations in patients before and 9 months after bariatric surgery.** A** Correlation between the differences in pre- and post-surgery whole-blood HbA1c and plasma magnesium concentrations (p = 0.024). **B** Correlation between whole-blood HbA1c and plasma magnesium concentrations in the pre- and post-surgery samples (p = 0.010)

Plasma concentrations of magnesium, copper, zinc and selenium from individual patients were compared to the respective HbA1c values measured either before or 9 months after surgery. No statistically significant correlation between pre-surgery values or between the post-surgery values were observed (where p≤0.05 is considered significant). A correlation between changes in HbA1c and magnesium concentration pre- and post-surgery was observed (p = 0.024; Fig.  4A). A significant correlation was also observed between HbA1c and plasma magnesium concentrations when both pre-and post-surgery values of these parameters across individuals were analysed together (p = 0.010; Fig. 4B). No correlations were observed between HbA1c and plasma concentrations of copper, zinc or selenium, when comparing differences in these parameters before or after surgery or when comparing both (pre- and post-surgery) sets of values together across the cohort. Again, whilst improved nutrition post-surgery may be a factor here, these data do indicate a potential relationship exists between plasma magnesium and glycaemia. In a previous study, we found plasma magnesium levels to be reduced in individuals with type-1 diabetes when compared to age-matched controls without diabetes (Sobczak et al. 2019). Subsequently, the reduction in plasma magnesium in this cohort was found to correlate with abnormalities in fibrin clot formation and lysis in the same cohort (Sobczak et al. 2020), potentially through a mechanism linked to dysglycaemia. Other studies linking magnesium to aberrant control of glycaemia include a meta-analysis involving 286,668 T2DM subjects, where an association between low dietary magnesium levels and T2DM was observed (Larsson and Wolk 2007). Similarly, in another study following 12,128 non-diabetic subjects over 6 years, an inverse relationship between serum magnesium concentration and incidence of T2DM amongst white (but not black) participants was reported (Kao et al. 1999). Magnesium supplementation has been shown to improve glycaemic control indicators, including whole-blood HbA1c levels, in individuals with type-2 diabetes (El Derawi et al. 2019). The link between plasma magnesium levels and glycation is likely the result of many factors. For example, magnesium is a cofactor of many enzymes involved in glucose metabolism (Paolisso et al. 1990) and plays important roles in insulin secretion and the actions of insulin (Barbagallo et al. 2003; Kostov 2019). It has been reported that low magnesium levels result in defective insulin receptor-associated tyrosine kinase activity, post-receptor impairment in insulin action and altered cellular glucose transport and utilisation, which promotes peripheral insulin resistance in type-2 diabetes (Kostov 2019).

## Conclusions

This study shows that bariatric surgery and the associated changes in lifestyle and diet (which include nutrient supplementation) lead to substantive weight loss and reduction in BMI and other anthropometric measures over a 9-month period. In addition, favourable outcomes relating to glycaemic status (as indicated by mean whole-blood HbA1c concentration) were apparent in this study. Modest improvement in plasma concentrations of magnesium and copper (and zinc/copper ratio) was observed 9 months after surgery. Although changes were generally within the normal range, improvement in the status of these nutrients would potentially impact positively on health aspects including appetite regulation through control of leptin levels, improvement in glucose metabolism and insulin action and a reduction in thrombotic risk. Whilst individual metals exhibited modest improvement, the mean zinc/copper ratio was much improved across the cohort (ideal zinc/copper ratio = 1.0) and would be expected to lead to an enhancement in the activity of enzymes utilising these metals for catalysis. In a few individuals, surgery and dietary/lifestyle changes led to abnormally high values of magnesium, copper or zinc, which if unchecked, have the potential to lead to additional health issues. These observations suggest that, while bariatric surgery and the subsequent nutritional treatments offered to the patients lead to improved health outcomes, treatment may be further optimised by monitoring plasma metal levels and the specific nutritional supplements taken by patients after bariatric surgery.

## Abbreviations

BMI: Body mass index
CV: Coefficient of variation
ICP-MS: Inductively-coupled plasma-mass spectrometry
ISIS-DS: Integrated sample introduction system and discrete sampler-3
N/A: Not applicable
NIST: National Institute of Standards and Technology, USA
RF: Radio frequency
